# Association of MRS-Based Vertebral Bone Marrow Fat Fraction with Bone Strength in a Human In Vitro Model

**DOI:** 10.1155/2015/152349

**Published:** 2015-04-19

**Authors:** Dimitrios C. Karampinos, Stefan Ruschke, Olga Gordijenko, Eduardo Grande Garcia, Hendrik Kooijman, Rainer Burgkart, Ernst J. Rummeny, Jan S. Bauer, Thomas Baum

**Affiliations:** ^1^Department of Diagnostic and Interventional Radiology, Technische Universität München, Ismaninger Straße 22, 81675 Munich, Germany; ^2^Department of Orthopedic Surgery, Technische Universität München, Ismaninger Straße 22, 81675 Munich, Germany; ^3^Philips Healthcare, Lübeckertordamm 5, 20099 Hamburg, Germany; ^4^Section of Neuroradiology, Technische Universität München, Ismaninger Straße 22, 81675 Munich, Germany

## Abstract

Bone marrow adiposity has recently gained attention due to its association with bone loss pathophysiology. In this study, ten vertebrae were harvested from fresh human cadavers. Trabecular BMD and microstructure parameters were extracted from MDCT. Bone marrow fat fractions were determined using single-voxel MRS. Failure load (FL) values were assessed by destructive biomechanical testing. Significant correlations (*P* < 0.05) were observed between MRS-based fat fraction and MDCT-based parameters (up to *r* = −0.72) and MRS-based fat fraction and FL (*r* = −0.77). These findings underline the importance of the bone marrow in the pathophysiology and imaging diagnostics of osteoporosis.

## 1. Introduction

Osteoporosis is a systemic skeletal disease characterized by reduced bone mineral density and microarchitectural deterioration, compromising bone strength and increasing risk of fractures [1]. Magnetic Resonance Imaging (MRI) is an ionizing radiation-free imaging modality, the application of which could be beneficial in the elderly population to monitor incidence, progression, and therapy of osteoporosis. Due to the lack of signal from the bone matrix in conventional MRI sequences, high-resolution MRI of the trabecular bone has been previously accomplished relying on the bone marrow signal surrounding the bone matrix [2, 3]. Bone marrow refers to the tissue occupying the cavities within the trabecular bone. The vertebral bodies, one of the most important fracture sites, are filled with red marrow, which is a mixture of haematopoietic red blood cells and fat cells. Despite recent progress [4, 5], direct high-resolution imaging of the trabecular bone has been limited on distal sites (e.g., radius, tibia, or calcaneus) and its application in red marrow regions remains technically challenging. Previous MRI investigations have studied alternative indirect measures of trabecular bone quantity and quality in red marrow regions, including approaches aiming to measure primarily bone marrow *T*_2_^∗^ [6, 7].

Another property of bone marrow, which has recently gained significant attention due to its potential association with bone loss pathophysiology, is its fat content [8–10]. It has been long known that bone marrow fat content increases with age [11]. MR investigations employing single-voxel Magnetic Resonance Spectroscopy (MRS) have recently shown the increase of vertebral bone marrow fat content with age in large scale in vivo studies [12, 13]. In addition to age-related change of bone marrow fat content, recent in vivo studies have shown that an increase in bone marrow fat content is associated with a decrease in bone mineral density (BMD) [7, 14–20]. Furthermore, there have been a limited number of studies analyzing transiliac bone biopsy samples showing an association between bone marrow adiposity and bone microstructure [21].

BMD remains the primary predictor of bone strength [22]. Additionally, it has been shown that imaging-based trabecular bone microstructure and texture parameters (using primarily computed tomography (CT) and its variants) can add significant information beyond BMD on predicting bone strength [23, 24]. Based on the negative association between BMD and vertebral bone marrow fat content, previous studies have recently proposed MRS-based bone marrow fat quantification as a potential noninvasive biomarker for prediction of fracture risk. However, many of these studies have used dual X-ray absorptiometry (DXA) for measurement of BMD, including therefore contributions from both the trabecular and the cortical bone compartments in the reported BMD measurements [7, 15–17]. In addition, there has been no previous groundwork on directly investigating the relationship between bone marrow fat content and biomechanical strength. Determining the relationship between bone marrow fat content and trabecular bone microstructure parameters would require sampling larger specimens than those obtained with biopsy. A multimodality ex vivo study in bone specimens combining CT and MRS measurements with biomechanical testing would be required to study the aforementioned relationships.

Therefore, the purpose of the present pilot study was to investigate the correlation of MRS-based proton density fat fraction with BMD/trabecular bone microstructure parameters obtained from multidetector CT (MDCT) measurements and bone strength determined from biomechanical testing, using human vertebral cadaveric specimens.

## 2. Materials and Methods

### 2.1. Specimens

Ten vertebrae between thoracic vertebrae 5 and 10 (T5–T10) were harvested from four fresh human cadavers (1 woman and 3 men; mean age ± standard deviation (SD) of 58 ± 12 years). Donors had no history of pathological bone changes other than osteoporosis (i.e., bone metastases, hematological, or metabolic bone disorders). The donors had dedicated their bodies for educational and research purposes to the local Institute of Anatomy prior to death, in compliance with local institutional and legislative requirements. Written informed consent was obtained from the donors. The study was reviewed and approved by the local institutional review boards.

The surrounding muscle, fat tissue, and intervertebral discs were completely removed from the vertebrae. Each vertebra was embedded in resin (Rencast Isocyanat and Polyol, Huntsman Group, Bad Säckingen, Germany) up to 2 mm above and below their vertebral endplates for the purpose of biomechanical testing. The resin fixation was performed with parallel alignment of the upper and lower endplate of the vertebrae with the outer surface of the resin chock to guarantee strict axial loading conditions of the vertebrae during the uniaxial biomechanical test. For the purpose of conservation, all vertebrae were stored in the freezer at 4° Celsius during the study and degassed in sodium chloride solution at least 3 h before imaging to prevent air artifacts. The vertebrae were sealed in vacuum plastic boxes filled with sodium chloride solution during imaging.

### 2.2. MDCT Imaging

Multidetector computed tomography (MDCT) images of the vertebrae were acquired by using a whole-body 256-row CT scanner (iCT, Philips Medical Care, Best, Netherlands). Scan parameters were a tube voltage of 120 kVp, a tube load of 585 mAs, an image matrix of 1024 × 1024 pixels, and a field of view of 150 mm. Transverse sections were reconstructed with a high-resolution bone kernel (YE). The real spatial resolution was 230 × 230 × 600 *μ*m^3^ as determined at *ρ*50 of the modulation-transfer-function. A dedicated calibration phantom (Mindways Osteoporosis Phantom, San Francisco, CA, USA) was placed in the scanner mat beneath the vertebrae.

### 2.3. MDCT Image Analysis

**Figure 1:**
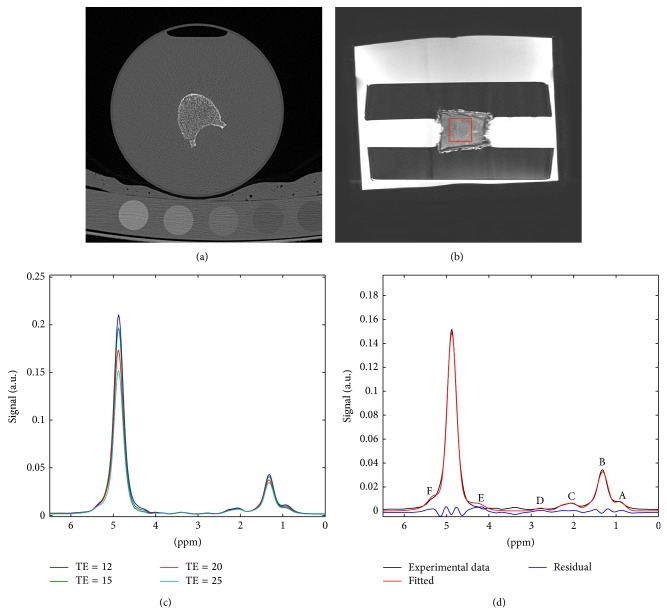
Representative images and spectra: (a) MDCT transverse image showing vertebral body and calibration phantom, (b) sagittal proton density-weighted MR image showing vertebral body and resin holders (the red box within the vertebra corresponds to the voxel position for the employed single-voxel MRS), (c) MR spectrum acquired at different echo times (12, 15, 20, and 25 ms), and (d) quality of spectrum fitting for the MR spectrum at TE = 25 ms (fat peaks A–F labeled along the spectral axis).

MDCT images were transferred to a remote LINUX workstation and loaded into an in-house developed program based on IDL (Interactive Data Language, Research Systems, Boulder, CO, USA). A radiologist performed all steps of the MDCT image analysis. First, the outer contour of each vertebra was segmented in the transverse sections. Then, transverse cross-sectional area was determined in each section to obtain the mean and minimum transverse cross-sectional area of each vertebra. Second, the twenty most central slices displaying the vertebra equidistant to its endplates were identified (Figure 1(a)). Similar to the MRS box (as outlined below), rectangular regions of interest (ROIs) with an area of 12 × 12 mm² were manually placed in the center of the vertebra in the selected twenty slices of the MDCT images. Lastly, ROIs were drawn in the phases of the calibration phantom in the MDCT images.

Mean BMD in the ROIs was calculated by converting the pixel attenuations in Hounsfield Units [HU] into BMD values in calcium hydroxyapatite [mg/cm^3^] by using the calibration phantom. Afterwards, MDCT images were binarized to calculate trabecular bone microstructure parameters. An optimized global threshold was applied to all MDCT images. Similar to previous studies, 200 mg/cm^3^ calcium hydroxyapatite was identified as optimized global threshold [25, 26]. Two morphometric parameters were calculated in the ROIs in analogy to standard histomorphometry using the mean intercept length method [27]: bone volume divided by total volume (BV/TV) and trabecular number (TbN; [mm^−1^]). In addition, fractal dimension (FD) as texture measurement of the trabecular bone microstructure was determined in the MDCT images using a box counting algorithm as previously described [26].

### 2.4. MR Imaging and Spectroscopy

The vertebrae were scanned on a 3 T whole-body scanner (Ingenia, Philips Healthcare, Best, Netherlands) using an 8-channel extremity coil. The MR exam consisted of two fat suppressed proton density- (PD-) weighted turbo spin-echo (TSE) sequences (one mimicking the sagittal anatomical orientation and one the axial anatomical orientation of the vertebrae) and one single-voxel MRS sequence. Sequence parameters for the TSE sequence were TE/TR = 40/3165 ms, TSE factor = 11, FOV = 130 × 130, 25 slices, slice thickness = 1.5 mm, and BW = 156.7 Hz/pixel.

Based on the specimen geometry outlined in the PD-weighted sequences (Figure 1(b)), a voxel was selected in the center of the vertebral body to perform single-voxel (12 × 12 × 12 mm^3^) MRS using a stimulated echo acquisition mode (STEAM) sequence with parameters: TR = 6 s (long TR to remove any *T*_1_ effects), TE = 12/15/20/25 ms (STEAM with short TEs to reduce J-coupling effects), 10 averages per TE, 2 phase cycles, 4096 data points, 5 kHz acquisition bandwidth, no water suppression, and no regional saturation bands. The voxel was positioned so that chemical shift displacement effects due to the finite bandwidth of the employed RF pulses used in the MRS voxel localization were minimized. Representative MR spectra at different TEs are shown in Figure 1(c).

### 2.5. MR Spectra Analysis

Spectra were fitted using Gaussian line shapes and frequency-based methods based on in-house MATLAB (MathWorks, Natick, MA) routines.  Figure 1(d) shows a typical bone marrow fat spectrum with fat peaks observed at spectral locations at 0.9, 1.30, 1.59, 2.00, 2.25, 2.77, 4.2, 5.19, and 5.31 ppm. The letters A, D, and E were assigned to peaks at 0.9 ppm (–(CH_2_)_*n*_–CH_3_), 2.77 ppm (–CH=CH–CH_2_–CH=CH–), and 4.2 ppm (–CH_2_–O–CO–), respectively. The letter B was assigned to the superposition of peaks at 1.30 ppm (–(CH_2_)_*n*_–) and 1.59 ppm (–CO–CH_2_–CH_2_–), the letter C was assigned to the superposition of peaks at 2.00 ppm (–CH_2_–CH=CH–CH_2_–) and 2.25 ppm (–CO–CH_2_–CH_2_–), and the letter F was assigned to the superposition of peaks at 5.19 ppm (–CH–O–CO–) and 5.31 ppm (–CH=CH–). Two water peaks were employed accounting for short and long *T*_2_^∗^ water components [28].

Peak fitting was performed by constraining the area of peaks E and F at a given ratio of peak A + B, based on the bone marrow triglyceride chemical structure determined previously [28]. A common linewidth was assumed for all fat peaks and independent linewidth values were fitted for the two water peaks, resulting in a total number of three linewidths as free variables. Fat peak locations were allowed to vary by ±0.05 ppm and water peak locations were allowed to vary by ±0.50 ppm. Peak fitting was performed for the spectra at individual TEs. *T*_2_ correction was then performed using nonlinear least squares fitting, assuming the same *T*_2_ relaxation time value for all fat peaks and a different value for the water *T*_2_ relaxation time. The derived proton density fat fraction was determined as the ratio of all the fat peaks (A, B, C, D, E, and F) area with the sum of all the fat peaks and the narrow (long *T*_2_^∗^) water peak area (i.e., excluding the broad-short *T*_2_^∗^ water peak area) [28].

### 2.6. Biomechanical Testing

The resin embedded vertebrae were fixed in a mechanical testing system (Wolpert Werkstoffprüfmaschinen AG, Schaffhausen, Switzerland). The biomechanical testing was performed similar to previous studies [26, 29]. Firstly, ten preconditioning cycles with uniaxial tension-compression up to a load between 10 N and 400 N with a rate of 5 mm/min were applied. Then, a monotonic, uniaxial compression was performed at the same rate. The load-displacement curve was recorded and vertebral failure load (FL) was defined as the first peak of the load-displacement curve with a subsequent drop of >10%.

### 2.7. Statistical Analysis

All statistical analysis was performed using SPSS (SPSS, Chicago, IL, USA). All tests were performed based on a 0.05 level of significance. Mean and SD of all MR-based (fat fraction), MDCT-based (BMD and trabecular bone microstructure parameters), and biomechanical testing-based (failure load of each individual vertebra divided by the minimum transverse cross-sectional area of each individual vertebra, i.e., normalized failure load) parameters were computed over the ten measured vertebra bodies. The Kolmogorov-Smirnov test showed for all parameters no significant difference from a normal distribution (*P* > 0.05). Therefore, correlations between the different parameters were computed with linear regression models and expressed as slope coefficient *B*, its standard error, correlation coefficient *r*, and its *P* value. Due to the relatively small sample size in this pilot study, the results were validated by bootstrapping techniques [30]. 1000 bootstrap samples of our data were determined by random sampling. Statistical results were expressed as bootstrap derived standard error of the coefficient *B* and *P* value of the regression model.

## 3. Results

Figure 1(c) shows the spectra acquired at different TEs on a vertebra, confirming a faster *T*_2_ relaxation for the water peak than for the fat peaks and verifying the need for *T*_2_ correction to derive a proton density fat fraction.  Figure 1(d) shows the experimentally measured spectrum and the fitted spectrum. There is a strong overlap between fat peaks E and F and the water peak, verifying the need for a constrained fitting of peaks E and F to achieve a reliable estimation of the water peak.

Table 1 summarizes the statistics (mean and SD values) of the main measured parameters. The proton density fat fraction ranged from 26% to 43% with a mean value of 32% and a SD of 5%. The transverse cross-sectional area of the vertebra ranged from 4.9 cm^2^ to 9.7 cm^2^ with a mean value of 6.5 cm^2^ and a SD of 1.5 cm^2^. The normalized FL ranged from 157 N/cm^2^ to 798 N/cm^2^ with a mean value of 442 N/cm^2^ and a SD of 251 N/cm^2^. Mean and SD values for BMD and trabecular microstructure parameters (BV/TV, TbN, and FD) are also listed in Table 1.

**Figure 2:**
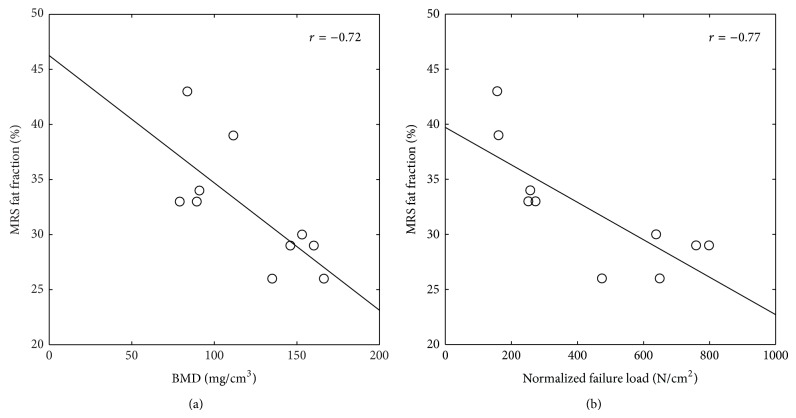
Relationship (a) between MRS proton density fat fraction and BMD and (b) between MRS proton density fat fraction and normalized failure load. The open circles represent the experimental results and the solid line shows the result of the linear regression.

Figure 2(a) shows a negative correlation of proton density fat fraction with BMD (*r* = −0.72, *P* = 0.045).  Figure 2(b) shows a negative correlation of proton density fat fraction with normalized FL (*r* = −0.77, *P* = 0.013). Proton density fat fraction also correlated with trabecular microstructure parameters, showing a trend close to statistical significance (*P* < 0.1) for BV/TV, TbN, and FD (Table 2).

Normalized FL showed strong correlations with BMD and all trabecular microstructure parameters with correlation coefficients ranging between 0.83 and 0.90 with *P* < 0.01 (Table 2). Bootstrap derived standard error of the coefficient *B* and *P* value of all regression models validated the obtained results (Table 2).

## 4. Discussion

The present in vitro study investigates, using human spine specimens, the relationship between MRS-based vertebral bone marrow proton density fat fraction, MDCT-based measures of BMD and trabecular microstructure, and biomechanical strength. A strong correlation was observed between biomechanical strength and BMD and between biomechanical strength and trabecular microstructure parameters. A correlation was also observed between bone marrow proton density fat fraction and biomechanical strength, providing for the first time a direct validation of the negative association between bone strength and bone marrow fat content.

The relationship between bone marrow fat content and BMD has been investigated in multiple previous studies [7, 14–21]. Specifically, previous works have shown statistically significant differences in bone marrow fat content between controls and subjects with osteopenia and osteoporosis (grouped based on their DXA-based *T*-score) [16–18]. It has been also shown that there is a negative correlation between fat content and DXA-based BMD [7, 15]. The limitation of DXA-based BMD measurements is that they include contributions from both trabecular and cortical bone components. It has not been until recently that trabecular BMD values based on quantitative computed tomography have been used to study the relationship between trabecular bone density and bone marrow fat content [16, 20]. The present study used thus MDCT-based trabecular BMD measures excluding the contributions from cortical bone compartments in the study between BMD and bone marrow fat content. In vivo measurement of vertebral trabecular microstructure parameters using MDCT would not be justifiable in clinical routine due to the need for high spatial resolution and the associated dose limitations (effective dose of estimated 3 mSv according to Graeff et al. [31]). That is why the acquisition of MDCT data ex vivo in specimens constitutes a meaningful step in establishing any association between trabecular microstructure parameters and bone marrow fat content.

The negative correlation between bone marrow fat content and bone density has been shown in multiple recent studies [7, 14–20]. Different mechanisms have been proposed for explaining this negative association, suggesting that bone marrow fat is not simply a “filler” of the bone matrix cavities [9]. Proposed mechanisms include a drift in mesenchymal stem cell differentiation that favors adipogenesis over osteoblastogenesis [32] or a direct effect of adipocytes on suppressing osteoblastogenesis [33]. In parallel, it is well known that bone density is positively correlated with biomechanical strength [22]. The present work shows a negative association between marrow fat content and biomechanical strength and provides to the best of our knowledge the first direct validation of the association between bone marrow fat content and biomechanical strength. Therefore, the present results complement the existing knowledge about the importance of bone marrow adiposity in understanding the pathophysiology of bone weakening and about the value of the MRS-based proton density fat fraction of bone marrow as an additional useful parameter in monitoring osteoporosis diagnosis, progression, and therapy. However, future larger scale studies would be necessary to understand whether marrow fat content can become a predictor of bone strength after correcting for BMD effects.

The present study uses single-voxel MR Spectroscopy to measure the bone marrow proton density fat fraction. Acquisitions with multiple TEs are performed to account for *T*_2_ effects and a constrained fitting approach is adopted taking into consideration the bone marrow triglyceride structure to avoid the inclusion of fat peaks E and F in the water peak signal [28]. The consideration of *T*_2_ effects and of the presence of the secondary fat peaks (E and F) overlapping with water peak aims for the extraction of a proton density fat fraction of bone marrow instead of a signal-weighted fat fraction reported in previous works [16–18]. By using this MR spectra analysis method and an appropriate parameter selection (long TR to remove any *T*_1_ effects and short TEs to reduce J-coupling effects), MRS using a stimulated echo acquisition mode (STEAM) as preformed in our present study can be successfully applied for in vivo bone marrow fat quantification as recently shown in the proximal femur and spine [28, 34].

Single-voxel MRS has been the technique most frequently used to measure fat content in different bone marrow regions, including the vertebral bodies and the proximal femur. However, single-voxel MRS provides very poor spatial resolution, which is an important limitation when applied in bone marrow regions with a spatial heterogeneous distribution of fat content [28]. Quantitative water-fat imaging techniques have recently been applied for measuring bone marrow proton density fat fraction with high spatial resolution in good agreement with single-voxel MRS after accounting for appropriate confounding factors [28, 35, 36].

The present study has some limitations. First, the sample size is relatively small. This relatively small sample size might be responsible for not reaching statistical significance (*P* < 0.1 but *P* > 0.05) when studying the relationship between bone marrow fat content and certain trabecular microstructure parameters (TbN and FD). Second, given the limited spatial resolution of MDCT, the MDCT-based measured values of the trabecular microstructure cannot depict the true trabecular structure. However, it has been previously shown that histomorphometric measurements as assessed with MDCT and micro-CT correlate significantly [37]. It has been also recently shown that the correlations of failure load versus trabecular bone microstructure parameters obtained with MDCT and high-resolution peripheral quantitative computed tomography (HR-pQCT) are not significantly different [26]. Third, the imaging-derived measurements are limited in the prediction of bone strength of an intact vertebra, since they do not account for the endplates, cortical shell, and posterior elements.

In conclusion, a negative relationship was observed between bone marrow proton density fat fraction and trabecular BMD and a negative relationship was observed between bone marrow proton density fat fraction and biomechanical strength. This in vitro study confirms the previously reported negative association between bone marrow fat content and bone density and provides the first direct ex vivo validation of a negative association between bone marrow fat content and bone strength. These findings underline the importance of the bone marrow in the pathophysiology and imaging diagnostics of osteoporosis.

## Figures and Tables

**Table 1:** Mean and SD values of experimental parameters measured with MRS (proton density fat fraction), biomechanical testing (failure load and normalized failure load), and MDCT (mean transverse cross-sectional area, BMD, and trabecular microstructure parameters).

Fat fraction (%)	32 ± 5
Mean transverse area (cm^2^)	6.5 ± 1.5
Norm. failure load (N/cm^2^)	442 ± 251
Failure load (N)	2580 ± 1093
BMD (mg/cm^3^)	121.5 ± 34.3
BV/TV (%)	38.8 ± 10.8
TbN (mm^−1^)	1.13 ± 0.11
FD	1.57 ± 0.07

**Table 2:** Linear regression models with correlation coefficient *r*, *P* value, slope coefficient *B*, and its standard error as well bootstrap derived standard error and *P* value of (i) MRS-based proton density fat fraction with normalized failure load (norm. FL), BMD, and trabecular microstructure parameters and (ii) normalized failure load (norm. FL) with MRS-based proton density fat fraction, BMD, and trabecular microstructure parameters.

	Norm. FL	Fat fraction	BMD	BV/TV	TbN	FD
Fat fraction						
*r*	−0.77		−0.72	−0.64	−0.62	−0.36
*P* value	**0.009**		**0.019**	**0.047**	0.059	0.314
Coefficient *B*	−3687		−0.001	−0.309	−0.296	−0.276
Standard error	1082		<0.001	0.132	0.134	0.257
Bootstrap standard error	1300		<0.001	0.112	0.145	0.187
Bootstrap *P* value	**0.013**		**0.045**	0.062	0.063	0.086
Norm. FL						
*r*		−0.77	0.90	0.88	0.89	0.83
*P* value		**0.009**	**<0.001**	**0.001**	**0.001**	**0.003**
Coefficient *B*		−3687	6.559	2056	2045	3111
Standard error		1082	1.156	386	380	730
Bootstrap standard error		1300	1.001	405	427	619
Bootstrap *P* value		**0.013**	**0.002**	**0.007**	**0.005**	**0.005**
